# Entwicklung einer App zur Unterstützung häuslicher Pflegeberatungsbesuche

**DOI:** 10.1007/s00391-023-02160-9

**Published:** 2023-02-01

**Authors:** Alexander Gabber, Sonja Heidenblut, Henrike Gappa, Tim La Guardia, Susanne Zank

**Affiliations:** 1 Department Heilpädagogik und Rehabilitation, Humanwissenschaftliche Fakultät, Universität zu Köln, Universitätsstr. 91, 50931 Köln, Deutschland; 2 Fraunhofer-Institut für Angewandte Informationstechnik FIT, Schloss Birlinghoven 1, 53757 Sankt Augustin, Deutschland

**Keywords:** Nutzerzentriertes Design, mHealth, Pflegedokumentation, Gruppenbasierte Expert:innen Walkthroughs, Interviews, User-centered design, mHealth, Care documentation, Group-based expert walkthroughs, Interviews

## Abstract

**Hintergrund:**

Die Pflegeberatungsbesuche nach § 37 Absatz 3 SGB XI (PBB) werden bisher uneinheitlich durchgeführt, und es mangelt an einer strukturierten Pflegedokumentation. Im Projekt INGE-integrate4care wurde eine App entwickelt, die die qualitätsgesicherte Durchführung von PBB unterstützen soll.

**Methode:**

Auf Basis eines nutzerzentrierten Designansatzes wurden 10 semistrukturierte Expert:inneninterviews geführt und mittels qualitativer Inhaltsanalyse nach Mayring ausgewertet. In gruppenbasierten Expert:innen-Walkthroughs mit 7 Teilnehmer:innen wurden Design, Inhalte und Funktionen der App diskutiert, Anforderungen in Feedbacklisten gesammelt und von Entwickler:innen digital umgesetzt.

**Ergebnisse:**

Die Interviewten berichteten, dass PBB heterogen seien, es individuelle Herangehensweisen der Pflegefachkräfte (PFK) gebe und sich dies in der Gestaltung der App wiederspiegeln solle. Wichtige Begutachtungsthemen für die App seien z. B. Inhalte des Neuen Begutachtungsassessments (NBA) und die Angehörigenbelastung. Funktionen wie die Empfehlung von Maßnahmen auf Basis dokumentierter Informationen seien wünschenswert. Hinderlich sei dagegen eine zu starre Einhaltung von Bearbeitungsschritten, da diese den Gesprächscharakter der PBB einschränken würden.

Das NBA und BIZA‑D wurden von den Expert:innen im Projekt als passende Basisassessments gewählt und an die PBB angepasst. Zur Unterstützung des Arbeitsablaufs wurden der flexible Zugriff auf Items, die Visualisierung des Pflegestatus je Kategorie sowie die Möglichkeit zu Auswahl und Nachverfolgung von Maßnahmen spezifiziert.

**Diskussion:**

Durch den nutzerzentrierten Designansatz konnte eine App entwickelt werden, die sich sowohl durch Flexibilität als auch Struktur auszeichnet. Das Tool wird mithilfe von Usability-Evaluationen und Fokusgruppen weiter optimiert.

## Abstract (en)

**Background:**

The in-home care consultation visits according to § 37.3 of the Social Security Statute Book XI have been carried out inconsistently so far and with a lack of structured care documentation. In the INGE-integrate4care project, an app was developed to support the quality assurance of visits.

**Method:**

Based on a user-centered design approach, 10 semistructured expert interviews were conducted and analyzed using qualitative content analysis according to Mayring. In group-based expert walkthroughs with 7 participants, the design, contents and functions of the app were discussed, requirements were collected in feedback lists and digitally implemented by developers.

**Results:**

Interviewees reported that visits are heterogeneous, that there are individual approaches and that this should be reflected in the design of the app. Important topics for the app were, e.g. contents of the NBA and the burden on caregivers. Functions such as the recommendation of measures on the basis of documented information are desirable. On the other hand, too stringent guidelines of the app would be an obstacle, as these would limit the conversational character of the visit.

The NBA and BIZA‑D were chosen by the experts in the project as suitable basic assessments and adapted to the visits. Flexible access to items, visualization of care status per category, and the ability to select and track measures were specified to support workflow.

**Discussion:**

The user-centered design approach enabled the development of an app that is characterized by both flexibility and structure. The tool will be further optimized with the help of usability evaluations and focus groups.

## Einführung und Hintergrund

Obwohl die Pflegeberatungsbesuche nach § 37 Abs. 3 SGB XI (PBB) eine wichtige Säule in der Sicherstellung der häuslichen Pflege sind, wird deren Potenzial aufgrund mangelnder Durchführungsstandards noch nicht hinreichend ausgeschöpft. Ziel des Projekts INGE-integrate4care ist es daher, eine App für Tablets zu entwickeln, die die PBB unterstützt und über ein implementiertes Assessment systematisch dokumentiert. Um die Appentwicklung auf fundiertem Wissen über die Bedürfnisse zukünftiger Endnutzer:innen aufzubauen, wurde ein nutzerzentrierter Designansatz gewählt.

### Pflegeberatungsbesuche nach § 37 Abs. 3 SGB XI

Etwa zwei Drittel der häuslichen Pflege in Deutschland werden von pflegenden Angehörigen (PA) geleistet [12]. PA steuern meist das gesamte Versorgungsgeschehen und sind dabei großen Belastungen ausgesetzt [1, 16]. Eine ungeeignete häusliche Situation, pflegerische Fehler oder fehlende Hilfeleistungen können lange unbemerkt bleiben [11]. Zudem können konflikthafte Beziehungen zwischen PA und Pflegebedürftigen zu psychischen Krisen oder missbräuchlichen Pflegesituationen führen [10]. Um die Qualität der informellen Pflege sicherzustellen, wurden die sog. PBB nach § 37 Abs. 3 SGB XI geschaffen, die sich an Pflegegeldempfänger:innen richten, die zu Hause von Angehörigen gepflegt werden. Ab Pflegegrad (PG) 2 wird hierbei die Gesamtsituation vor Ort halbjährlich und gesetzlich verpflichtend von qualifizierten Pflegefachkräften (PFK) begutachtet, und ggf. Maßnahmen zur Stabilisierung der häuslichen Pflegesituation empfohlen. Ab PG 4 müssen die Besuche vierteljährlich stattfinden. Bei PG 1 oder Bezug von Pflegesachleistungen kann die Beratung halbjährlich freiwillig in Anspruch genommen werden [8]. Die Dauer der PBB unterscheidet sich z. T. stark zwischen den Bundesländern und sogar einzelnen Pflegediensten. Zwischen Aug. 2019 und Dez. 2020 dauerten die meisten PBB in Bayern zwischen 30 und < 45 min, wobei in dieser Zeit aufgrund der Pandemie viele PBB telefonisch stattgefunden haben [9]. Die Vergütungen in Bayern sind an die Durchführungszeit geknüpft. In anderen Bundesländern wird „eine auf Landesebene vereinbarte Punktzahl mit dem individuellen Punktwert des Pflegedienstes multipliziert“ [9].

In den PBB geht es darum, „subjektive und objektive Beratungsbedarfe explizit zu machen und diese Problem- oder Risikobereiche zu identifizieren“ [4]. Solche Problembereiche sind z. B. die Selbstständigkeit und der Unterstützungsbedarf des Pflegebedürftigen, der Pflegezustand, das Wohnumfeld, soziale Kontakte sowie Herausforderungen des/der PA [4, 8].

Aufbau und Durchführung der PBB wurden schon seit ihrer Einführung diskutiert und viele der Kritikpunkte, wie z. B. der „Kontrollcharakter“ der Besuche oder die Anforderungen an eine PFK später aufgegriffen [7]. Es existieren jedoch immer noch keine einheitlichen Standards für die Begutachtung [11]. Das Potenzial der Einsätze wird dadurch nicht hinreichend ausgeschöpft [2]. Zwar gibt es gesetzliche bzw. fachliche Qualitätsanforderungen wie die Empfehlungen nach § 37 Absatz 5 SGB XI zur Qualitätssicherung der PBB [8] sowie erste Versuche zur Entwicklung von Qualitätsstandards [7], bisherige wissenschaftliche Ansätze, die Dokumentation zu strukturieren, beschränken sich jedoch auf die Zusammenfassung geeigneter Assessmentinstrumente oder die Entwicklung eines papierbasierten Leitfadens [4, 11]. Papierbasierte Dokumentation kann aber zeitaufwendig, repetitiv und ggf. ungenau sein [20]. Digitale Pflegedokumentation hingegen ermöglicht eine weniger fehleranfällige, vollständigere und stärker auf den Patienten ausgerichtete Dokumentation [14, 18].

### Entwicklung der INGE-App

Die INGE-App wurde von einem interdisziplinären Team aus Software-Entwickler:innen, Versorgungsforscher:innen, Usability-Expert:innen und PFK entwickelt. Herzstück der App ist ein Instrument, das eine systematische Begutachtung der häuslichen Versorgungssituation ermöglicht.

Da der individuelle Unterstützungsbedarf der Klient:innen im Fokus steht, wurde dem INGE-Assessment der im zweiten Pflegestärkungsgesetz definierte Pflegebedürftigkeitsbegriff zugrunde gelegt. Dieser wurde in dem von Wingenfeld et al. 2011 entwickelten Neuen Begutachtungsassessment (NBA) operationalisiert, das zum Zwecke der Pflegegradeinstufung durch den MDK Funktions- und Selbstständigkeitseinschränkungen in den Teilbereichen Mobilität, Kognition und Kommunikation, Verhalten, Selbstversorgung, Gestaltung des Alltagslebens und sozialer Kontakte sowie Bewältigung von krankheits- oder therapiebedingten Anforderungen und Belastungen erhebt [19]. Darüber hinaus soll die INGE-App die Belastung von Pflegepersonen erfassen, wie in den neuen Empfehlungen zur Qualitätssicherung nach § 37 Absatz 5 SGB XI der PBB angeregt wird [8]. Hierzu soll ein Instrument Verwendung finden, das Belastung möglichst multidimensional misst.

Bei der Entwicklung müssen jedoch auch die speziellen Anforderungen des Pflegeberatungssettings im Fokus stehen, d. h. vor allem Offenheit der Gesprächssituation, knappe Zeitressourcen und die Verknüpfung des erfassten Unterstützungsbedarfs mit Empfehlungen zu individuell passenden Maßnahmen und Hilfsmitteln zur Stabilisierung der Pflegesituation [8, 11].

Um dies gewährleisten zu können, wurden die zukünftigen Endnutzer:innen von Anfang an in den Gestaltungsprozess miteinbezogen.

## Methode

**Figure 1:**
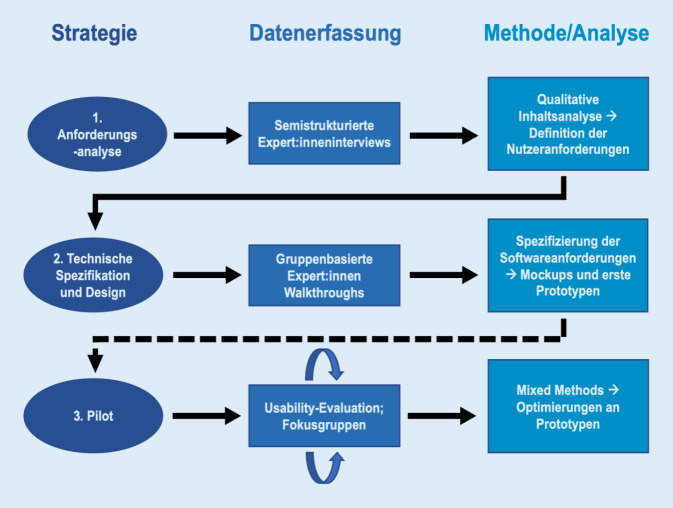

Die Entwicklung der App erfolgte in 3 Schritten (Abb. 1). Theoretische Grundlage bildeten die Ansätze der menschzentrierten Gestaltung aus DIN EN ISO 9241-210:2020-03 [3]. Im ersten Schritt wurden Interviews mit Pflegeberatungsexpert:innen geführt, um ein erstes Verständnis für Nutzungskontexte, Herausforderungen sowie relevante Inhalte und Funktionen des zu entwickelnden Instruments zu erhalten. Aus daraus abgeleiteten Nutzeranforderungen und Nutzungsszenarien wurde ein erstes Vorführmodell (Mockup) entworfen, das im zweiten Schritt über die von Følstad 2007 [5] entwickelte Methode des gruppenbasierten Expert:innen-Walkthrough regelmäßig angepasst wurde. Darauf aufbauend entstanden erste digitale Prototypen. Im dritten Schritt wird eine erweiterte Usability-Evaluation erfolgen. Dieser Artikel beschreibt die Stufen 1 und 2 des Prozesses.

### Stufe 1: Anforderungsanalyse

#### Durchführung

Die semistrukturierten Expert:inneninterviews wurden zwischen dem 03.12.2019 und dem 02.03.2020 zunächst persönlich, nach Ausbruch der Pandemie per Videotelefonie geführt. Sie wurden mit Einverständnis der Interviewten aufgenommen und anonymisiert transkribiert. Die Interviews dauerten im Schnitt 61,2 min bei einem Range von 39 bis 103 min.

Die Interviewten wurden u. a. danach gefragt, wie Begutachtungen und Empfehlungen während der PBB ablaufen und durch eine App unterstützt werden könnten, welche Begutachtungsinhalte enthalten sein sollten und welche Nachteile durch die Nutzung eines solchen Tools entstehen könnten.

### Stufe 2: Gruppenbasierte Expert:innen-Walkthroughs

#### Durchführung

Auf Grundlage der Interviewauswertungen wurden erste Nutzeranforderungen definiert und in Zusammenarbeit mit PFK Nutzungsszenarien erstellt, die zur Entwicklung von Mockups dienten. Die Mockups stellten Entwürfe des Designs und der vorgesehenen Workflows dar, z. B. zur Dokumentation einer vorgeschlagenen Maßnahme. Die Mockups und später auch die ersten Prototypen wurden mithilfe von gruppenbasierten Expert:innen-Walkthroughs evaluiert, d. h., typische Aufgaben von Endnutzer:innen wie z. B. die Dokumentation der Aussage eines:r Pflegebedürftigen wurden Schritt für Schritt ausgeführt und hinsichtlich intuitiver und aufgabenangemessener Gestaltung beurteilt. Die Expert:innengruppe war interdisziplinär zusammengesetzt, und jede:r Teilnehmer:in urteilte aus Sicht des individuellen Domänenwissens. Im Unterschied zur sonst üblichen Vorgehensweise bei Walkthroughs wurden Probleme nicht nur identifiziert, sondern in der Gruppe auch Lösungsvorschläge erarbeitet und für Entwickler:innen als Softwareanforderungen festgehalten [6].

### Datenauswertung

Die Interviews wurden mittels inhaltlich strukturierender Inhaltsanalyse nach Mayring [13] in MAXQDA 2020 (VERBI Software GmbH, Berlin, Deutschland) analysiert und ausgewertet. Aus dem Material wurden induktive Haupt- und Unterkategorien herausgearbeitet, paraphrasiert und gebündelt. Die Kodierung erfolgte durch 3 Mitarbeiter:innen jeweils unabhängig voneinander und wurde anschließend bis zur Einigung diskutiert.

### Genehmigung der Ethikkommission

Die Studie wurde von der Ethikkommission der Humanwissenschaftlichen Fakultät der Universität zu Köln als unbedenklich eingestuft.

## Ergebnisse

### Rekrutierung und Stichprobe

Die Rekrutierung der Interviewten erfolgte über webbasierte soziale Berufsnetzwerke. Es nahmen 10 Pflegeberatungsexpert:innen teil. Teilnehmende der gruppenbasierten Expert:innen-Walkthroughs waren 7 regelmäßige Mitglieder des Projektkonsortiums (Tab. 1).

**Table 1:** 

Stichprobe	Beruf *n* (%)	Alter MW (SD)	Geschlecht *n* (%)	
			w	m
**Interviews**	10 (100)	50,6 (±6,6)	6 (60)	4 (40)
*Interventionsforscher*	1	–	–	–
*Pflegeberaterin*	1	–	–	–
Professor Pflegemanagement	1	–	–	–
Pflegedienstleitung	2	–	–	–
Pflegegutachter:in	2	–	–	–
Pflegedozentin	1	–	–	–
Pflegeberater:in mit sonstiger Zusatzqualifikation	2	–	–	–
**Gruppenbasierte Expert:innen-Walkthroughs**	7 (100)	50,1 (±11,8)	4 (57,1)	3 (42,9)
*Usability-Expert:in*	2	–	–	–
*Versorgungswissenschaftler:in*	2	–	–	–
*Pflegedienstleitung*	1	–	–	–
*Pflegeberater:in*	2	–	–	–

### Anforderungsanalyse

Ergebnisse der inhaltlich strukturierenden Inhaltsanalyse waren 4 Hauptkategorien: Erfahrungen mit dem PBB, wünschenswerte Inhalte, wünschenswerte Funktionen sowie hinderliche Nutzungsfaktoren.

#### Erfahrungen mit dem PBB

Hier berichteten die Interviewten, dass Beratungssituationen heterogen sind und deutliche Unterschiede im Zeitaufwand zwischen Erst- und Folgebesuchen bestehen (*n* = 5). Zudem wurde deutlich, dass PFK individuelle Herangehensweisen an die Durchführung des PBB haben und sich dies in der Gestaltung der App widerspiegeln sollte.

#### Wünschenswerte Inhalte

Die Interviewten beschrieben z. B. die Mobilität (*n* = 6), Kognition (*n* = 2), Barrierefreiheit in der Wohnung (*n* = 2) und Selbstversorgung des Pflegebedürftigen (*n* = 2) sowie eine Erfassung der Angehörigenbelastung (*n* = 2) als relevante Inhalte für das Tool. Ein geeignetes Basisinstrument sei das NBA (*n* = 2).

#### Wünschenswerte Funktionen

Die Befragten nannten hier eine übersichtliche Darstellung des Pflegestatus über ein Ampelsystem (*n* = 2), eine Notizfunktion (*n* = 2), eine individuelle Maßnahmenempfehlung (*n* = 5) sowie eine Hilfsmitteldatenbank (*n* = 7). Auch eine Nachverfolgung des Pflegeverlaufs solle über das Tool möglich sein (*n* = 5).

#### Hinderliche Nutzungsfaktoren

Hier wurde geäußert, dass der bisherige Arbeitsablauf und die Offenheit des Beratungsgesprächs durch zu stringente Vorgaben, wie z. B. die obligatorische Verwendung von Assessments, gefährdet werden könnten (*n* = 8). Außerdem wurden Datenschutzprobleme (*n* = 3) sowie eine geringe Technikaffinität (*n* = 2) mancher PFK als hinderlich für die Nutzung genannt.

### Entwicklung des Prototyps

#### Inhalte der App: Instrument

Zur systematischen Erfassung des häuslichen Pflegesettings wurden 2 Assessmentinstrumente als Grundlage verwendet: das NBA und das Berliner Inventar zur Angehörigenbelastung-Demenz (BIZA-D) [17, 19].

Das NBA wurde in den Walkthroughs so adaptiert, dass zentrale Aspekte für die Begutachtung der PBB enthalten waren. Es wurden 5 Kategorien (Mobilität, kognitive und kommunikative Fähigkeiten, Verhaltensweisen und psychische Problemlagen, Selbstversorgung und Gestaltung des Alltagslebens und soziale Kontakte) übernommen und in ihrem Wortlaut leicht verändert, um sie an das Format des Tablets anzupassen (Tab. 2). Als weitere Kategorie wurde der Gesundheitsstatus aufgenommen, der neben Items aus der 6. NBA-Kategorie (Bewältigung von und selbstständiger Umgang mit krankheits- oder therapiebedingten Anforderungen und Belastungen) auch andere Themen enthält. Diese sind: Schmerzstatus, pflegerelevante Diagnosen, Medikamente, Allergien, Probleme mit dem Hautzustand, Bewusstsein, Körperbau, Blutzucker und Atmung. Zudem wurden Stammdaten, Daten zum Wohnumfeld und Notfallinformationen (z. B. Vollmachten) integriert. Die Skalierung der Kategorie „Verhalten“ wurde überarbeitet, da die PFK eine Unterscheidung zwischen „nie“ und „selten“ als relevant für die PBB erachteten. Aus der 4‑stufigen Skala: (0) nie oder sehr selten bis täglich (3) wurde die 5‑stufige Skala: (1) nie bis (5) sehr häufig (mindestens einmal pro Tag).

**Table 2:** 

Instrument	Kategorie	Beispiel	Skala	Items (INGE)	Items (NBA)
NBA	Kognition & Kommunikation	Mitteilung elementarer Bedürfnisse	(1) Fähigkeit vorhanden bis (4) Fähigkeit nicht vorhanden	7	11
	Mobilität	Umsetzen können	(1) Selbstständig bis (4) unselbstständig	5	6
	Selbstversorgung	An‑/Auskleiden können	(1) Selbstständig bis (4) unselbstständig	12	13
	Verhalten	Wie häufig müssen Sie als Pflegeperson bei Ängsten eingreifen?	(1) Nie bis (5) sehr häufig (mindestens einmal pro Tag)	8	13
	Soziales Umfeld	Gestaltung des Tagesablaufs	(1) Selbstständig bis (4) unselbstständig	3	6
	Gesundheitsstatus	Medikation	Selbstständige Einnahme; Hinweise; Wirkstoff; Grund; Stärke; Tageszeit; Form	12	16
BIZA‑D	Belastung durch persönliche Einschränkungen	Haben Sie das Gefühl, dass Sie zu wenig Zeit für Hobbys/Interessen haben?	(In den letzten zwei Wochen) (1) nie bis (5) immer	5	9
	Belastung durch kognitive Einbußen	Haben Sie das Gefühl, dass der/die Betroffene sich nichts merken kann?	Ja, nein ≠nein: Wie sehr belastet Sie das? (1) gar nicht bis (5) stark	3	4
	Belastung durch negative Selbstbewertung	Haben Sie das Gefühl, dass Sie die Pflege nicht im Griff haben?	(In den letzten zwei Wochen) (1) nie bis (5) immer	3	3

Skalierungen in den NBA-Kategorien wurden um die Antwortoption „unklar“ ergänzt

*BIZA‑D* Berliner Inventar zur Angehörigenbelastung-Demenz, *NBA* Neues Begutachtungsassessment

Für jede Kategorie wird, wie im NBA, ein Status ermittelt, der Einschränkungen in der Selbstständigkeit bzw. der Fähigkeiten des/der Pflegebedürftigen abbildet. Dieser wird über ein vierfarbiges Ampelsystem visualisiert: grün: unabhängig oder keine Einschränkungen, gelb: geringe Einschränkungen, orange: erhebliche Einschränkungen, rot: starke Einschränkungen bis zum vollständigen Verlust. Da die INGE-App als Leitfaden zur Unterstützung der Dokumentation und Beratung dient, gibt das Instrument hierbei keine Gesamteinschätzung über die Sicherstellung der Pflege aus, sondern die Entscheidung darüber unterliegt der Expertise der PFK.

Aus dem BIZA‑D wurden 3 Subskalen zur Angehörigenbelastung aufgenommen, die mit typischen Risiken in Pflegesituationen einhergehen, nämlich dem Depressionsrisiko für informell Pflegende, dem Risiko von Gewalt in der Pflege und dem Risiko der Institutionalisierung des Pflegebedürftigen [15, 17]. Diese wurden ebenfalls in einem Ampelsystem visualisiert: grün: geringes Risiko, gelb: moderates Risiko, orange: hohes Risiko, rot: extremes Risiko.

#### Funktionen und Interfacedesign der App

**Figure 2:**
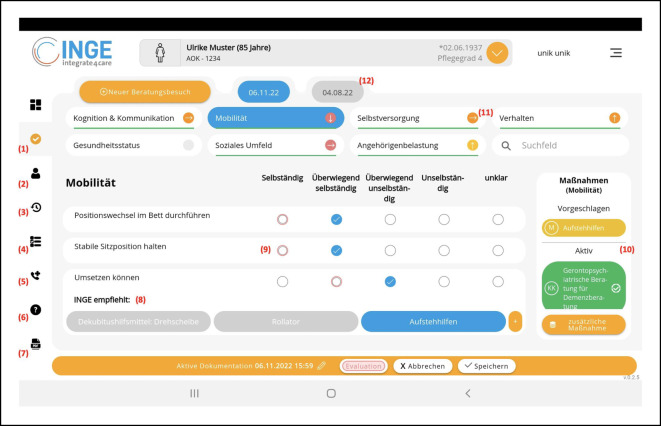

Neben den inhaltlichen Aspekten wurden Funktionen in die App integriert, die eine Begutachtung unterstützen sollen (Abb. 2). Dabei wurde berücksichtigt, dass PBB als offenes Gespräch geführt werden. Die App wurde daher so gestaltet, dass ein flexibler Zugriff auf Kategorien und Items erlaubt ist, zwischen denen schnell hin- und hergewechselt werden kann. Über visuell hervorgehobene Item-Ratings kann in Folgebesuchen mit einem Blick die Bewertung des Vorbesuchs überprüft und mit der Ist-Situation verglichen werden. Ein Ampelsystem zeigt den aktuellen Pflegestatus an, Veränderungen zum Vorbesuch werden in Form eines Pfeilsystems visualisiert. Über die Historie können alle vorhergehenden Besuche eingesehen werden. Zudem ermöglicht die App das Erstellen, digitale Signieren und Versenden von GKV-Nachweisformularen als PDF.

In die App wurde ein Katalog von rund 200 Maßnahmen aus dem Hilfsmittelverzeichnis des GKV-Spitzenverbandes nach §139 SGB V integriert, die den Klient:innen in Bereichen empfohlen werden können, in denen unter Berücksichtigung der Assessmentergebnisse und der Einschätzung der PFK Verbesserungspotenzial besteht. So zeigt die App z. B. ab einer leichten Einschränkung im „Erkennen von Risiken und Gefahren“ die 3 Empfehlungen „Hausnotruf, Herdsensor und Ortungssystem“ an. Der Status ausgewählter Maßnahmen kann jeweils nachverfolgt werden (z. B. „vorgeschlagen“, „bewilligt“, „abgelehnt“ etc.). PFK haben jedoch immer auch die Möglichkeit, aus dem gesamten Katalog zu wählen und auch neue Maßnahmen hinzuzufügen.

## Diskussion

Die INGE-App wurde entwickelt, um PBB nach § 37.3 SGB XI zu unterstützen. Begutachtungsthemen, verknüpfte Maßnahmen sowie Designaspekte wurden von den Projektpartner:innen in Zusammenarbeit mit PFK erarbeitet und fortlaufend angepasst. Durch die Implementierung des NBA und BIZA‑D ermöglicht die App eine systematische Erfassung des Pflegestatus. Die Verknüpfung mit einem Maßnahmenkatalog unterstützt die PFK dabei, individuell zugeschnittene Anregungen zu geben, die zu einer Stabilisierung der Pflegesituation beitragen können. Die Darstellung von Verläufen unterstützt zudem einen dynamischen Einfluss auf Akutsituationen und ein schnelles Ergreifen von Gegenmaßnahmen.

Durch die Einbindung von Endnutzer:innen wurde deutlich, dass die Funktionsweise der App den Kontext der PBB berücksichtigen muss. Die App ermöglicht daher eine individuelle Herangehensweise der PFK im Rahmen eines offenen, vertrauensvollen Gespräches. Dabei unterstützt die App eine strukturierte Dokumentation, gibt den PFK jedoch keine vollstandardisierte Erfassung der Situation vor, da eine solche der Heterogenität der PBB nicht gerecht werden würde.

Mit dem NBA und dem BIZA‑D wurden Instrumente in das Assessment integriert, die sich in anderen Kontexten als valide erwiesen haben und unter Berücksichtigung von Expert:innenbefragungen und Walkthroughs an die Anforderungen des PBB angepasst wurden. Die limitierten zeitlichen Ressourcen der PFK in den PBB wurden z. B. durch eine nicht-obligatorische Eingabe eines Großteils der Items berücksichtigt. Dies wirft die Frage auf, wie sich eine solche zugestandene Flexibilität auf das Eingabeverhalten auswirkt. Erste Analysen hierzu laufen bereits und sind Bestandteil zukünftiger Untersuchungen.

Die Gebrauchstauglichkeit des Instruments wird derzeit von 9 PFK im Feld überprüft, die etwa 500 App-gestützte PBB durchführen. Die ersten Ergebnisse aus Fokusgruppe und Usability-Tests weisen darauf hin, dass das Tool den Pflegestatus aus Sicht der PFK gut und übersichtlich erfasst und sich intuitiv bedienen lässt. Besonders die Items aus dem BIZA‑D, die eine Thematisierung der bisher wenig erfassten Angehörigenbelastung ermöglichen, wird von den Nutzer:innen als Bereicherung empfunden. PFK berichten jedoch auch, dass die mit der App durchgeführten Erstbesuche aufgrund des ausführlichen Assessments geringfügig mehr Zeit in Anspruch nahmen, dafür aber Folgebesuche durch die unterstützende Verlaufsansicht und digital archivierten Informationen aus den Vorbesuchen effizienter durchgeführt werden konnten.

Die App wurde in einem fortlaufenden Prozess entwickelt, bei dem die Rückmeldungen der Endnutzer:innen sukzessive zu einer Verbesserung des Tools beigetragen haben. Aufgrund der Komplexität häuslicher Pflegesituationen ist es dabei sinnvoll, die Inhaltsvalidität und Gebrauchstauglichkeit auf der Grundlage weiterer PBB zu überprüfen und zu optimieren. So wird die Integration von Themen wie sensorischer Einschränkungen, Sturzrisiko und Schluckstörungen diskutiert. Zudem wird die Ausführlichkeit, in der einige Bereiche wie z. B. die Angehörigenbelastung abgefragt wird, reduziert, um den zeitlichen Ressourcen der PFK besser gerecht zu werden.

Darüber hinaus wurden potenzielle Vorteile der INGE-App mit weiteren Akteuren wie PA, Ärzt:innen sowie Pflegefachkräften und Pflegedienstleiter:innen der stationären Pflege diskutiert. Hierbei ging es insbesondere um die Möglichkeit, den Informationsaustausch über Schnittstellen effizienter zu gestalten. Die Analysen hierzu sind Bestandteil zukünftiger Untersuchungen. Mit PA sind zudem noch Befragungen zur Wahrnehmung und zur Zufriedenheit mit den App-gestützten PBB geplant.

### Schlussfolgerung

Die nutzerzentrierte Entwicklung eines App-Prototypen ist ein Versuch, die PBB digital zu unterstützen und zu strukturieren. Seit Mitte 2021 wird die App von PFK in PBB angewandt und weiter evaluiert, um Inhalte, Funktionen und Design optimal an die Bedürfnisse der Endnutzer:innen anzupassen.

## Fazit für die Praxis

Die INGE-App ermöglicht:

- effiziente Dokumentation und Digitalisierung von Pflegeberatungsbesuchen nach § 37 Abs. 3 SGB XI (PBB),
- schnell erfassbare Information über den letzten Stand der häuslichen Pflegesituation durch visuelle Hervorhebung von Item-Ratings aus letztem PBB und Verlaufsdarstellung,
- Unterstützung der Empfehlungen durch vorselektierte Maßnahmen,
- Generierung eines digitalen GKV-Nachweisformulars.
